# Fatigue and Corrosion Fatigue Behaviour of Brazed Stainless Steel Joints AISI 304L/BAu-4 in Synthetic Exhaust Gas Condensate

**DOI:** 10.3390/ma12071040

**Published:** 2019-03-29

**Authors:** Anke Schmiedt-Kalenborn, Lars Andree Lingnau, Matthias Manka, Wolfgang Tillmann, Frank Walther

**Affiliations:** 1 Department of Materials Test Engineering (WPT), TU Dortmund University, Baroper Str. 303, D-44227 Dortmund, Germany; andree.lingnau@tu-dortmund.de (L.A.L.); frank.walther@tu-dortmund.de (F.W.); 2 Institute of Materials Engineering (LWT), TU Dortmund University, Leonhard-Euler-Str. 2, D-44227 Dortmund, Germany; matthias.manka@tu-dortmund.de (M.M.); wolfgang.tillmann@tu-dortmund.de (W.T.)

**Keywords:** brazed joint, AISI 304/BAu-4, fatigue, corrosion fatigue, synthetic exhaust gas condensate

## Abstract

As brazed stainless steel components in service often have to withstand cyclic loads in corrosive environments, the corrosion fatigue properties of brazed joints have to be characterised. Application-relevant corrosion fatigue tests in corrosive media are extremely rare for brazed joints and cyclic deformation curves are barely investigated. In this study, fatigue tests of brazed AISI 304L/BAu-4 joints were performed in air and synthetic exhaust gas condensate K2.2 according to VDA 230-214. The fatigue behaviour of the brazed joints was compared to properties of the austenitic base material. Strain, electrical, magnetic, temperature and electrochemical measurement techniques were applied within fatigue and corrosion fatigue tests to characterise the cyclic deformation and damage behaviour of the brazed joints. It was found that the fatigue strength of 397 MPa at 2 × 10^6^ cycles was reduced down to 51% due to the superimposed corrosive loading. Divergent microstructure-related damage mechanisms were identified for corrosion fatigue loadings and fatigue loadings of specimens in the as-received and pre-corroded conditions. The investigations demonstrate the important role of corrosive environments for the mechanical performance of brazed stainless steel joints.

## 1. Introduction

Brazing techniques are used for a reliable and economic production of joint structures for various applications in the automotive and chemical industry as well as for components for power generation. During the operation, brazed structures are commonly mechanically loaded within corrosive environments. Hence, the effect of corrosion on the tensile and fatigue test performance of brazed joints is of great interest for the product development. In the literature, mechanical and corrosion properties of brazed joints of stainless steels have been described [1,2]. However, fatigue tests of pre-corroded specimens as well as application-relevant corrosion fatigue tests in corrosive media are extremely rare for brazed joints and the cyclic deformation curves are barely described [3]. Initial studies of the authors on the corrosion fatigue performance of brazed AISI 304L/BNi-2 joints using a nickel-based filler metal in a synthetic condensate are already available [4,5]. It is expected that the results cannot be transferred to other material combinations such as high-strength and more corrosion-resistant brazed stainless steel joints.

Brazed joints with gold-base filler metals show a great corrosion resistance and high mechanical strength at room temperature [6,7,8]. In this study, the fatigue behaviour of brazed AISI 304L/BAu-4 joints was analysed and compared to the properties of the austenitic base material. For metastable austenites, the deformation-induced formation of martensite and the influence on the fatigue behaviour are presented in numerous publications [9,10,11,12,13,14,15]. It is well known that the martensitic transformation leads to a cyclic hardening and increases the lifetime [16,17]. In contrast, a self-heating due to the cyclic loading can inhibit the martensitic transformation and reduce the lifetime [9,16,18].

Micro-compositional differences across the brazing seam are reported in the literature [19,20] and known to promote a local corrosive attack due to the formation of localised galvanic cells in different media [4,20,21,22]. Thus, the fatigue properties of the brazed specimens are expected to be significantly degraded due to the corrosive environments. The present study focused on the corrosion fatigue behaviour of the brazed AISI 304L/BAu-4 joints. Thereby, in situ corrosion fatigue tests were performed in the synthetic exhaust gas condensate K2.2, with a chemical composition according to the VDA (Verband der Automobilindustrie) test sheet 230-214 [23], using a corrosion cell to simulate realistic damage mechanisms. To compare the corrosion fatigue behaviour with the effect of a long-time corrosion, pre-corroded brazed specimens were additionally investigated. The pre-corrosion was executed for up to six weeks, as defined in [23]. First results of tensile and fatigue tests of the brazed AISI 304L/BAu-4 joints in the as-received condition and after pre-corrosions, obtained by means of the digital image correlation (DIC) technique, were published in earlier studies by the authors [8,24,25]. Parts of the fractographic analyses were used for the current study to compare damage mechanisms generated by corrosion fatigue loading and fatigue loading before and after a long-term pre-corrosion. 

The aim of this study was to characterise the fatigue and corrosion fatigue properties of brazed stainless steel joints with the gold-base filler metal BAu-4 using an innovative test setup, because the application-relevant fatigue behaviour in corrosive media is unknown. In comparison to brazed joints with the nickel-based brazing alloy BNi-2, which were investigated earlier, high mechanical strengths and an improved corrosion resistance were expected. The cyclic deformation behaviour of the brazed AISI 304L/BAu-4 joints was compared to the properties of the austenitic base material with a deformation-induced formation of martensite. The degradation of the fatigue properties due to the loading in synthetic exhaust gas condensate was quantified and corrosion fatigue damage mechanisms were analysed, taking the microstructure and micro-compositional differences at the brazing seam into account. For the first time, the fatigue and corrosion fatigue properties of the brazed stainless steel joints using the gold-based filler metal BAu-4 were compared to brazed joints using the nickel-based brazing alloy BNi-2. 

## 2. Materials and Methods

### 2.1. Materials

**Figure 1:**
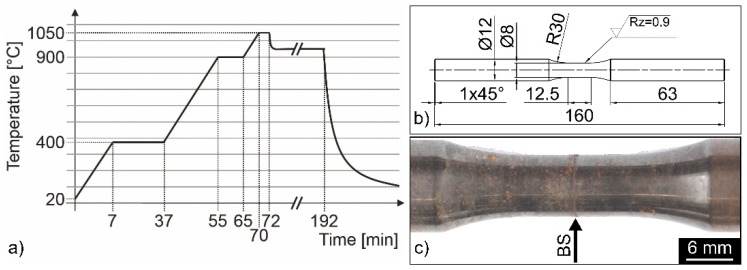
(**a**) Time–temperature curve for the brazing process [25]; (**b**) specimen geometry (all dimensions in mm) [8]; and (**c**) six weeks pre-corroded brazed specimen [8].

The austenitic stainless steel AISI 304L (X2CrNi18-9; 1.4307; DIN EN 10088-3, Thyssenkrupp Schulte GmbH, Essen, Germany) and the gold-base brazing alloy BAu-4 (Au 827, Au82Ni18, Umicore Technical Materials, Hanau, Germany), which was applied as a 50 µm amorphous brazing foil, were used to manufacture the brazed butt joints. The chemical composition of the base material is listed in Table 1. For the brazing process, a vacuum furnace was used with a vacuum of 2 × 10^−5^ to 8 × 10^−5^ mbar. Figure 1a shows the temperature–time curve with a brazing temperature of 1050 °C and a dwell time of 2 min. Subsequently, a heat treatment for 2 h at 950 °C and a free cooling to room temperature were conducted. 

The brazed cylindrical bars were used to manufacture fatigue and corrosion fatigue test specimens according to the technical drawing in Figure 1b. A few brazed specimens were pre-corroded according to the VDA 230-214 [23], which is relevant for exhaust gas-carrying components, with the aim to compare the results to the investigations on brazed AISI 304L/BNi-2 joints [4,5]. The pre-corrosion was performed for up to six weeks with periods of semi-immersion, drying and a steam phase and a weekly heating for 5 h at 400 °C. The alternating corrosion exposure mainly leads to a local circumferential corrosive attack in the diffusion zones (Figure 1c) [8].

**Figure 2:**
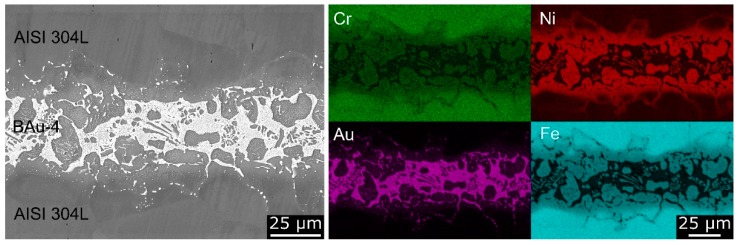
Microstructure of brazing seam (SEM and BSE) with element distribution maps (SEM and EDS).

The microstructure of the brazing seam, which consists of a mixture of mainly two phases, was analysed by means of scanning electron microscopy (SEM, Tescan Mira 3 XMU, TESCAN GmbH, Dortmund, Germany) with a back-scattered electron (BSE) detector (Figure 2). According to the element distribution maps, in contrast to the iron-, nickel- and chromium-rich grey phase, the white phase mainly contained gold. As a result of interalloying effects during the brazing process iron, chromium and silicon dissolved into the braze alloy and gold penetrated into the base material, as also shown in [3,26]. A reduction of the chromium, gold and nickel contents within the diffusion zones was detected using an energy dispersive X-ray spectroscopy (EDS). Micro-compositional differences at the interfaces and diffusion zones of welded and brazed joints are known to promote the formation of localised galvanic cells, leading to local corrosive attacks [20,21,22].

### 2.2. Experimental Setup and Procedure

**Figure 3:**
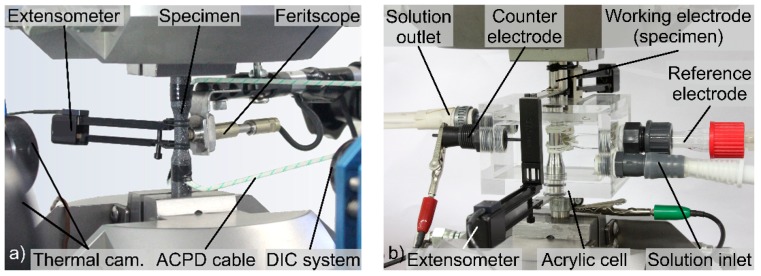
Experimental setup for: (**a**) fatigue tests [8]; and (**b**) corrosion fatigue tests.

Fatigue and corrosion fatigue tests were performed with a servo-hydraulic testing system (F = 100 kN) utilising a frequency f = 10 Hz, a stress ratio R = 0.1 and sinusoidal load–time functions. The stress ratio R = 0.1 is usually used for the design of brazed exhaust gas heat exchangers. The maximum stress σ_max_ was constantly controlled during the tests up to the limited number of cycles N = 2 × 10^6^. During the fatigue tests of eight specimens in air, an extensometer with a gauge length of 12.5 mm (GL12.5) for strain measurements of up to ±40% was used. In addition, the material response during the fatigue tests was evaluated with electrical, temperature and magnetic measuring techniques. The deformation-induced change in electrical voltage ∆U was determined in the gauge length with a crack growth monitor for alternating current (AC) potential drop measurements. Here, the change in voltage ∆U was calculated based on the initial data from the test beginning. The change in magnetic portion ∆ζ in the metastable austenitic base material close to the brazed joints was detected with a feritscope sensor to characterise the deformation-induced formation of martensite. The thermal imaging camera was used to measure the temperatures at the gauge length (T_1_) and at the specimen’s shafts (T_2_ and T_3_). Afterwards, the temperature change was calculated by ∆T = T_1_ − 0.5 ∙ (T_2_ + T_3_). Figure 3a shows the experimental setup with thermal imaging cameras (thermoIMAGER TIM 400, Micro-Epsilon Messtechnik GmbH, Ortenburg, Deutschland), a feritscope (Fischerscope MMS, Helmut Fischer GmbH, Sindelfingen, Deutschland), a crack growth monitor (CGM-7, Matelect LTD, Harefield, UK) and a DIC system (Q400, Limess GmbH, Krefeld, Deutschland). For the DIC analyses [8,24,25], a speckle pattern was painted on the surface of the specimens using an airbrush system.

Corrosion fatigue tests of eight specimens were performed in the synthetic exhaust gas condensate K2.2, as defined in the VDA test procedure 230-214 [23]. The synthetic condensate with a pH of 3.5 consisted of 0.039 mL/L formic acid (3.9 × 10^−8^), 0.035 mL/L acetic acid (3.5 × 10^−8^) and 1650 mg/L NaCl (1.65‰) dissolved in water. The experimental setup for corrosion fatigue tests, including an in situ corrosion cell, is shown in Figure 3b. Strain values were measured integrally with an extensometer with knife edges positioned at the shafts of the specimens, allowing a qualitative assessment of the cycle deformation behaviour within a gauge length of 62 mm. As total strain values up to 40% can be obtained for austenitic stainless steel joints, reliable local strain measurements at the brazing seam in the media using an extensometer with drop-down extensions [4] cannot be used. The gauge lengths are relevant for the comparison of the strain values of fatigue tests in air and in corrosive media. For electrochemical measurements, a standard three-electrode system was integrated into the self-designed acrylic cell. Thereby, a silver chloride (Ag/AgCl) reference electrode and a glassy carbon counter electrode were used. The brazed specimen served as the working electrode. A potentiostat enabled the continuous measurement of the change in the open circuit potential E_OCP_ of the brazed specimens, which is a result of deformation- and damage-induced microstructural changes. The fracture surfaces were investigated using SEM with a BSE detector and a secondary electron (SE) detector to characterise the microstructure-related fatigue and corrosion fatigue damage mechanisms.

## 3. Results and Discussion

### 3.1. Fatigue Behaviour in Air

To evaluate the fatigue damage behaviour, the material reactions of brazed specimens under cyclic loadings in air were recorded using various measurement techniques. Hence, the progressions of the total mean strain ε_m,t_, the total strain amplitude ε_a,t_, the loss energy density w, the change in temperature ΔT and the change in AC voltage ΔU are presented as functions of the load cycles N in the following. The loss energy density w describes the areas of the hysteresis loops and is calculated by w = ∮ σ dε. 

**Figure 4:**
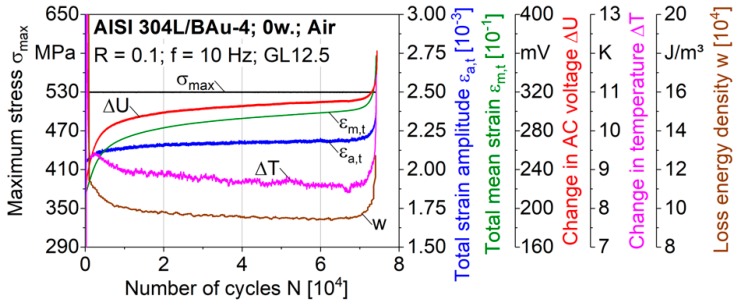
Constant amplitude test of a brazed specimen, performed in air.

The characteristic curves for a fatigue test with a constantly controlled maximum stress σ_max_ = 530 MPa are presented in Figure 4 a linear grit to improve the correlation of the different techniques for the fatigue state close to failure. During the initial fatigue stage up to about 10^4^ cycles, a considerable total mean strain ε_m,t_ up to 23% was reached. The increase of the total mean strain was a result of the directional accumulation of plastic strains due to the stress ratio of R = 0.1. In addition, the loss energy density w decreased regressively due to cyclic hardening effects. Therefore, the hysteresis loops were continuously shifted to higher strain values, while the size of the hysteresis loop areas and the absorbed energy per cycle decreased. As an increase of the magnetic portion of Δζ = 6 vol.% was detected with the feritscope after 3000 cycles, a deformation-induced phase transformation to martensite was the reason for the cyclic hardening in the initial stage. The amount of the formed martensite is known to increase with increasing plastic deformations. Thus, a decreasing ratcheting strain rate within the first fatigue stage was expected to contribute to the degressive trend of the martensite formation and to a regressive change of the loss energy density. Within the following saturation stage, the cyclic deformation curves indicated a steady strain rate with slowly progressing ratcheting fatigue and cyclic hardening effects. Within the third stage, starting at about 5000 cycles before failure at N_f_ = 7.4 × 10^4^, progressively increasing ε_m,t_, ε_a,t_ and w values indicated a crack propagation until failure.

For the overall fatigue life, the ΔU curve corresponded well with the total mean strain. The direct relation of the curves may be used for a quantitative analysis of the cyclic deformation behaviour of brazed joints in future studies. In addition, there was a direct relation of the cyclic hardening behaviour and the specimen’s temperature, because 95% of the loss energy density w is known to dissipate into heat. At the life fraction N/N_f_ = 0.5, a change in temperature of ΔT = 8.7 K, with an absolute temperature within the gauge length T_1_ = 42.8 °C, was determined. As the maximum change in temperature ΔT was below 20 K, a significant influence of the self-heating on the martensite formation could be excluded [11]. Consequently, temperature and electrical measurements are well applicable for brazed AISI 304L/BAu-4 joints to characterise ratcheting fatigue and cyclic hardening effects.

A comparable evolution of ratcheting strains is described and explained for austenitic stainless steels in [10,12,14]. During the first fatigue stage, the increasing density of the mobile dislocations and the formation of dislocation cells lead to a degressive increase of the total mean strain. The saturation is expected to be a result of a stable configuration of the newly generated dislocations in the base material [14]. During the third stage, the progressive increase indicates rapid dislocation activities and crack propagations until failure [12].

**Figure 5:**
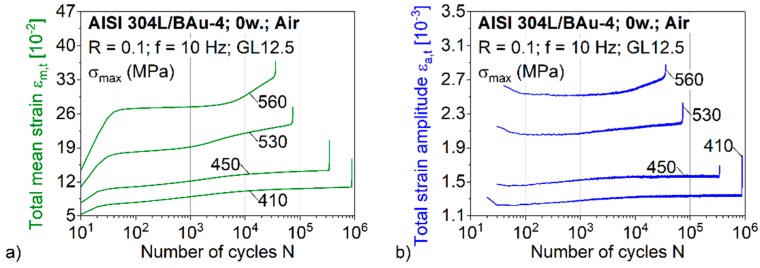
(**a**) Total mean strain curves; and (**b**) total strain amplitude curves for brazed specimens, tested in air.

**Figure 6:**
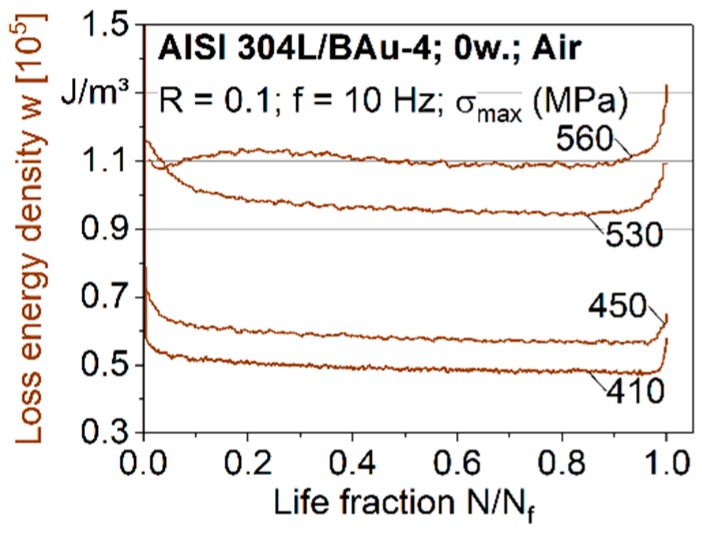
Loss energy density curves for brazed specimens, tested in air.

The cyclic deformation curves, determined in four fatigue tests with constantly controlled maximum stresses σ_max_ within the range of 410–560 MPa, are presented in Figure 5 and Figure 6. A greater accumulation of ratcheting strains at the beginning of the test and pronounced ratcheting strain rates in all three characteristic fatigue stages could be detected for higher stress levels of the brazed AISI 304L/BAu-4 joints (Figure 5a). For all specimens, the ratcheting strain rate decreases considerably during the saturation stage compared to the rate at the test beginning. The highest stress level of σ_max_ = 560 MPa led to total mean strains of approximately 28% within the saturation stage. The accompanying increase of the total strain amplitudes with increasing fatigue stresses is visualised in Figure 5b. The decreasing σ_m_ and σ_a_, combined with decreasing ε_m,t_ and ε_a,t_ lowered the total damage and hence extended the fatigue life.

For austenitic stainless steels, the degree of deformation during cyclic loading, depending on the applied stresses, has already been investigated and the results published [9,10,12,13,14]. During cyclic loading, the highest density of mobile dislocations in the base material is expected to be obtained at the time of the maximum tension loading [12]. As there are no compressive stresses and no reverse plastic deformations during unloading, formed dislocations cannot be annihilated. Consequently, a net storage of mobile dislocations in the base material after each loading cycle is expected, which is more pronounced for higher maximum stresses [12,14]. Furthermore, inelastic ratcheting strains during fatigue loading lead to a reduction of the cross-sectional area of the brazed specimens and to an increase in true stresses [12]. This effect is also expected to be more pronounced for higher fatigue stresses, leading to higher ratcheting rates, especially in the third fatigue stage [12]. The described decrease of the total damage and the extension of the fatigue life for the brazed joints with decreasing σ_m_ and σ_a_, combined with decreasing ε_m,t_ and ε_a,t_, was also reported for the base material [13,14].

With increasing fatigue stresses, the cyclic hardening in the first and second stage was more pronounced, although the respective decrease of w always showed a higher gradient at the test beginning in comparison to the second steady state (Figure 6). The third stage constantly signified cyclic softening due to fatigue damage, crack propagation and failure. Presenting the results in a linear grit of the life fraction N/N_f_ enabled evaluating the relative proportions of the three deformation stages during the fatigue lives. For higher stresses, cyclic hardening at the beginning of the test and cyclic softening effects close to failure were present for a higher proportion of life. For the highest stress level σ_max_ = 560 MPa, a cyclic hardening within the first cycles could be determined, followed by a cyclic softening and a subsequent secondary cyclic hardening. For lower stresses, a cyclic softening of the brazed joints could not be shown with the areas of hysteresis loop.

An earlier phase transformation to martensite and a higher martensite formation rate for higher fatigue stresses and ratcheting strains are also described for austenitic stainless steels [9,14,27]. It is well documented in the literature that the deformation-induced formation of martensite is significantly influenced by the chemical composition of the metastable austenitic stainless steels and the loading condition, which influences the degree of deformation and the deformation temperature [10,15,28]. A self-heating of the specimens due to cyclic loading with constant amplitudes leads to changes in temperature ΔT < 20 K. Thus, a significant effect on the formation of martensite can be neglected [11].

The evolution of the loss energy density w of the brazed joint, tested at the highest stress level σ_max_ = 560 MPa, could be correlated to test results of the austenitic stainless steel [10]. Within the first cycles, the formation and interaction of new dislocations were expected to lead to a cyclic hardening, followed by a cyclic softening due to the increasing mobility of the dislocations and the formation of a dislocation structure. The subsequent secondary cyclic hardening can be explained by a deformation-induced martensitic transformation [9,10]. Consequently, the cyclic deformation behaviour of the brazed stainless steel joints using the gold-based filler metal BAu-4 is characteristic for the metastable austenitic base material [9,14]. A detailed analysis of the fatigue damage behaviour with respect to microstructural changes in the brazing seam requires additional experiments and metallographic investigations. 

**Figure 7:**
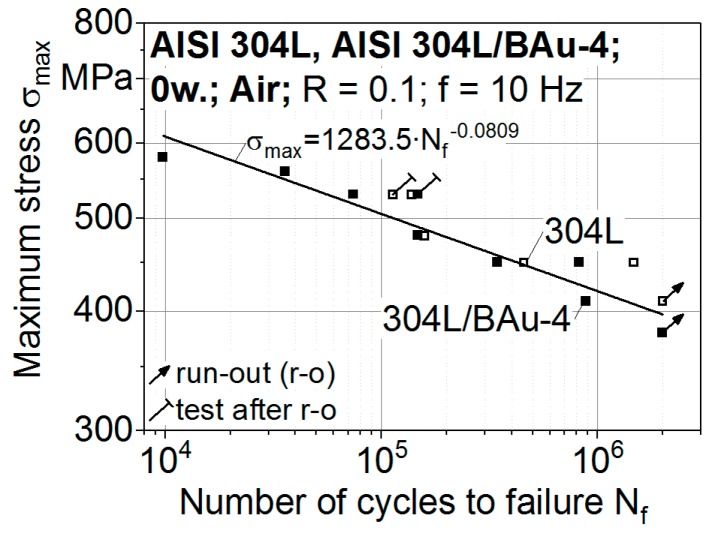
Results of constant amplitude tests of brazed specimens and AISI 304L specimens, performed in air.

The results of eight fatigue tests were analysed by relating the controlled maximum stress levels σ_max_ to the number of cycles to failure N_f_ in a diagram with a double logarithmic scale (Figure 7). In addition, results of the AISI 304L base material from earlier studies [5] are presented. The S-N curve for the brazed joints is described with the fit line σ_max_ = S_D_ ∙ (N_D_/N_f_)^1/k^ according to Basquin, with the coefficient of determination R^2^ = 0.9018. Thereby, the fatigue strength of 397 MPa at 2 × 10^6^ cycles and the gradient k = 12.4 were calculated [8]. The results of the base material [5] were positioned within the scatter range of the experimental data of the brazed joints. It can be concluded that the fatigue strength up to 2 × 10^6^ cycles of the stainless steel AISI 304L is not significantly reduced due to the brazing process used. 

### 3.2. Corrosion Fatigue Behaviour in a Synthetic Exhaust Gas Condensate

**Figure 8:**
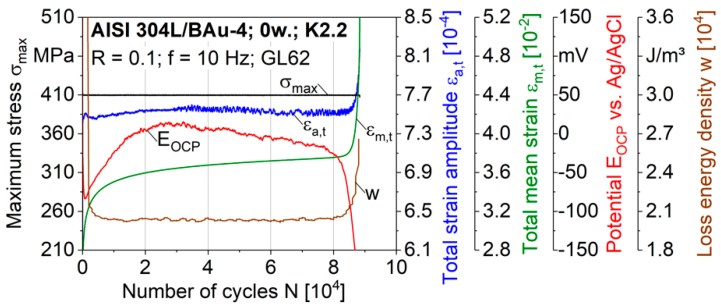
Constant amplitude test of a brazed specimen, performed in the synthetic condensate K2.2.

The results of fatigue tests with superimposed corrosion fatigue loadings, performed in situ with σ_max_ = 410 MPa in a synthetic exhaust gas condensate K2.2, are shown in Figure 8. The measured values of the total strain amplitude ε_a,t_, total mean strain ε_m,t_, loss energy density w and the electrochemical open-circuit potential E_OCP_ are visualised in dependence on the load cycles N. Comparable to the fatigue tests in air, a continuous increase of the total mean strain ε_m,t_ with three characteristic stages was detected due to ratcheting effects. In contrast, ε_a,t_ remains at a constant level up to 5000 cycles prior to the failure at N_f_ = 8.8 × 10^4^. Within the first stage, a decrease of w indicates cyclic hardening effects due to the deformation-induced phase transformation to martensite. The strain values, which were measured integrally with an extensometer with knife edges positioned at the shafts of the specimen, only allow a qualitative assessment of the cyclic deformation behaviour. For local strain measurements at the brazing seam in the condensate, the use of drop-down extensions at the knife edges of the extensometer will be optimised in future studies. 

At the very beginning of the cyclic loading, a sharp decrease of E_OCP_ down to −84 mV was observed. Up to 3 × 10^4^ cycles, E_OCP_ was shifted to higher values, whereby the rate of change decreased. Subsequently, a continuous cathodic shift of E_OCP_ was determined during the steady strain rate stage until up to approximately 8.3 × 10^4^ cycles. Finally, a significant decrease of E_OCP_ indicated the failure of the specimen. 

The evolution of E_OCP_ (Figure 8) wass expected to be mainly influenced by dissolution and repassivation mechanisms, which depend on the cyclic deformation and damage behaviour of the brazed joints. The abrupt decrease of E_OCP_ at the beginning of the test indicated a quick rupture of the passive layer of the stainless steel due to the first loading. As a result of the high ratcheting strain rate within the first stage, additional active material surface was exposed to the electrolyte. Here, the increasing E_OCP_ was attributed to the repassivation of the active stainless steel surface. Despite the decreasing ratcheting strain rate in the steady state, leading to a smaller formation rate of the active surface, a cathodic shift was determined. The sharp cathodic shift within the third stage was correlated with the cyclic softening due to fatigue damage, crack propagation and failure. 

In the literature, a continuous cathodic shift of E_OCP_ of stainless steels, mechanically loaded in a medium, is also described as a result of a steady rupture of the passive film [29,30,31]. The rupture leads to an anodic dissolution of the active material surface below the passive oxide layer, which is exposed to the electrolyte. In general, a formation of a passive film can lead to a gradual increase of the E_OCP_ [30]. The destruction and formation of an oxide layer during cyclic loading in a medium depend on various parameters such as the temperature, the test frequency, loading waveform and stress ratio [32]. By increasing the load and the loading speed, the degree of the cathodic shift of the E_OCP_ of the austenitic stainless steel AISI 304 is known to be more pronounced because of a reduced time for the repassivation [4,30].

In the following, possible reasons for the cathodic shift during the steady state are discussed. To evaluate the E_OCP_ of brazed joints, electrochemical potential differences, which occur due to different compositions of the gold-base filler metal and the austenitic stainless steel, have to be taken into consideration. Furthermore, local sensitised areas and micro-compositional differences can occur at the interfaces and diffusion zones, as already reported for welded and brazed joints [4,21,22]. The resulting localised galvanic cells in the area of the brazing seam generate active local regions, which are prone to local corrosive attacks [20,21]. In addition, the thermal expansion mismatch between the austenitic stainless steel and the gold-base filler metal during the brazing process may have generated residual stresses in the area of the brazing seam [2]. Residual stresses are also known to increase the susceptibility of environmentally assisted cracking with respect to crack initiation and propagation, as shown for weldments [21]. Thus, the cathodic shift during corrosion fatigue loading of brazed joints is expected to be influenced by the fatigue crack initiation and propagation as well as by the passivation kinetics, passive film thickness [33] and local galvanic corrosion effects [21,22]. The fracture position and fracture behaviour as well as the deformation-induced formation of martensite may also affect the corrosion fatigue behaviour. In this study, the possible reasons for the cathodic shift during the steady state were not further investigated.

**Figure 9:**
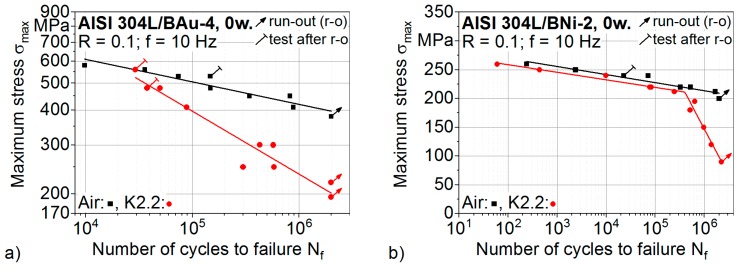
Results of constant amplitude tests, performed in air and in synthetic condensate K2.2: (**a**) brazed AISI 304L/BAu-4 specimens of the current study; and (**b**) brazed AISI 304L/BNi-2 specimens from earlier studies [34].

The controlled maximum stress levels in relation to the numbers of cycles to failure N_f_, determined in eight fatigue and eight corrosion fatigue tests of brazed specimens, are given in Figure 9a. The S-N curve of the specimens, tested in the synthetic condensate, is described with a fit line in the double logarithmic scale. A fatigue strength of 202 MPa at 2 × 10^6^ cycles and a steeper gradient of k = 4.4 was determined with R^2^ = 0.9334. Thus, the fatigue strength at 2 × 10^6^ cycles of specimens tested in air was significantly reduced down to 51% due to the superimposed corrosive loading. As corrosion mechanisms are time-dependent [32], longer test durations are known to increasingly affect the fatigue properties of the brazed joints. For the design of the brazed components, the effect of the test frequency on the corrosion fatigue behaviour has to be taken into consideration.

The authors investigated stainless steel joints brazed with the nickel-base filler metal BNi-2 in earlier studies [34]. The S-N curves of the brazed AISI304L/BNi-2 joints determined for fatigue and corrosion fatigue loadings are presented in a semi-logarithmic scale in Figure 9b. In contrast to the high-strength brazed AISI304L/BAu-4 joints as well as the applied austenitic base material, a significant reduction of the fatigue properties down to 50% is observed. At 2 × 10^6^ cycles, a fatigue strength of 210 MPa is obtained in air. The number of cycles to failure N_f_ of brazed AISI304L/BNi-2 joints cyclically tested with maximum stresses above 210 MPa is reduced with a factor of approximately 5 in the synthetic condensate K2.2. Smaller stresses increasingly influence the fatigue behaviour due to longer test durations. A fatigue strength of 90 MPa was determined in the synthetic condensate for 2 × 10^6^ cycles. Thus, a reduction of the fatigue strength down to 43% due to the superimposed corrosive loading has to be taken into consideration for the brazed AISI304L/BNi-2 joints [34]. 

When comparing the percentage values of 51% (AISI304L/BAu-4) and 43% (AISI304L/BNi-2), the corrosion fatigue behaviour of the brazed joints using the gold-base filler metal seems to be improved. Considering the absolute values, the fatigue strength at 2 × 10^6^ cycles was reduced by 195 MPa for the AISI304L/BAu-4 joints and by 120 MPa for the AISI304L/BNi-2 joints. Therefore, a greater degradation of the fatigue properties due to the loading in synthetic condensate was determined for the high-strength and corrosion-resistant brazed joints with the gold-base filler metal. A comparison of the microstructure-based corrosion fatigue damage mechanisms of brazed AISI304L/BAu-4 and AISI304L/BNi-2 joints will be presented in future studies. 

### 3.3. Fractographic Analysis

**Figure 10:**
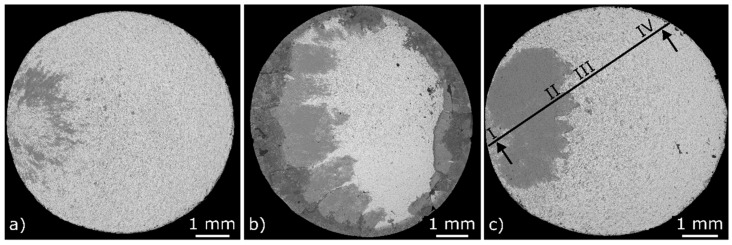
Fracture surfaces of brazed specimens: (**a**) in the as-received condition, cyclically tested in air; (**b**) after six weeks of pre-corrosion, cyclically tested in air [8,25]; and (**c**) in the as-received condition, cyclically tested in the synthetic condensate K2.2 (SEM, BSE).

The fatigue and corrosion fatigue damage mechanisms were analysed based on fracture surfaces and polished sections using SEM. Thereby, the dark-grey stainless steel and the light-grey filler metal BAu-4 could be distinguish with a BSE detector. The damage mechanisms of the brazed specimens, cyclically tested in air before and after pre-corrosions according to the VDA test procedure 230-214 [23], are presented in earlier studies by the authors [8,24,25]. In air, the fatigue crack was initiated on the left side of the fracture surface, as presented in Figure 10a. The origin of the fatigue crack was an imperfection close to the surface within the brazing seam. Such an imperfection with a free solidified surface of the filler metal appeared light-grey on the fracture surface and was described by the authors in detail in [8]. After initiation, the crack propagated alternatingly within the brazing seam, the interfaces and the base material close to the diffusion zone. In this context, dark-grey areas can be seen next to the light-grey areas in the crescent-shaped fatigue fracture area. The final fracture with pronounced deformation characteristics was detected in the centre of the light-grey brazing seam [8,25].

After the long-time pre-corrosion, a circumferentially corrosive attack in combination with pits in the area of the brazing seam was detected on the fracture surface (Figure 10b). The local corrosive attack in the area of the diffusion zones is a result of micro-compositional differences at the brazing seam, which lead to the formation of local galvanic cells. The corroded diffusion zones serve as a crack initiation zone and lead to numerous dark-grey fatigue fracture areas. Here, the cracks mainly propagated within the base material. The final fracture occurred in the light-grey brazing seam [8,25]. 

**Figure 11:**
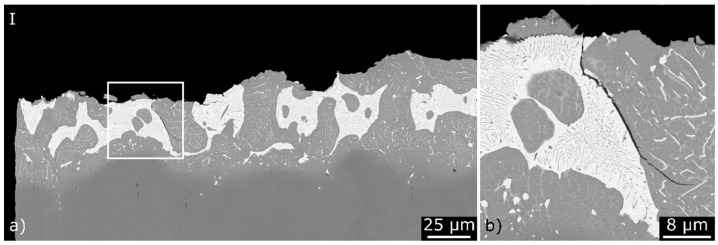
(**a**) Polished sections of fatigue specimens, tested in the synthetic condensate K2.2 in the zone of the fatigue crack initiation; and (**b**) a detailed view (SEM and BSE).

The fracture surface of a brazed specimen after corrosion fatigue loading in the synthetic condensate K2.2 with σ_max_ = 410 MPa for a test duration of 2.5 h, is shown in Figure 10c. A single fatigue crack initiation can be identified on the left side of the top-view. The polished section of the crack initiation zone shows a fracture in the centre of the brazing seam that commonly occurred at the transition between the gold-rich and nickel-rich phases (Label I, Figure 11a). A secondary crack between these phases can be seen in more detail in Figure 11b.

**Figure 12:**
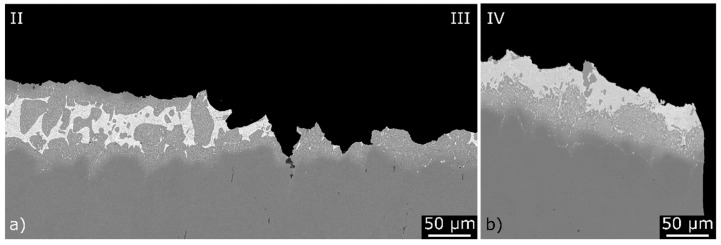
Polished sections of fatigue specimens, tested in the synthetic condensate K2.2: (**a**) at the transition from the fatigue fracture area to the forced fracture area; and (**b**) in the forced fracture area (SEM and BSE).

In accordance with the as-received and pre-corroded specimens tested in air, the fatigue fracture (Label II) is mainly located in the base material close to the brazing seam and the final fracture (Labels III and IV) occurred within the brazing seam (Figure 12a,b). A polished cross-section of the area, where the crack position changes from the base material to the brazing seam, is shown in Figure 12a. In contrast to the earlier investigations on brazed AISI 304L/BNi-2 joints [4], corrosive products were not detected on the fracture surface of specimens that were cyclically tested in the medium. Therefore, a repassivation of the active stainless steel fracture surface was expected. Such corrosion fatigue damage mechanisms have also been described for non-brazed austenitic stainless steels [35,36].

The fracture characteristics for the fatigue fracture and final fracture areas are quite comparable for fatigue and corrosion fatigue loaded brazed specimens. The fatigue fracture areas of specimens, cyclically tested in the synthetic condensate (Figure 10c), were more pronounced than the fatigue fracture areas of specimens tested in air (Figure 10a). It was assumed that the brazing seam was susceptible to environmentally assisted cracking and, therefore, the fatigue crack was initiated in the medium at lower fatigue stresses. Lower stresses in combination with the medium promoted the fatigue crack propagation in the base material, which led to a larger fatigue fracture area when compared to specimens tested in air. 

The fractographic investigations enabled a comparison of the damage mechanisms of brazed AISI 304L/BAu-4 joints caused by corrosion fatigue loading and fatigue loading after a long-term pre-corrosion. After pre-corrosions, the local corrosive attack in both diffusion zones reduced the remaining cross-section area and facilitated several fatigue crack initiations (Figure 10b). In contrast, isolated fatigue crack initiations occurred during testing in the synthetic condensate K2.2 and the fatigue crack initiation was environmentally assisted. Finally, the application-relevant corrosion fatigue behaviour of brazed joints could not be sufficiently characterised by fatigue tests of pre-corroded joints. Corrosion fatigue tests in the synthetic condensate need to be additionally taken into consideration.

## 4. Conclusions

Fatigue tests and corrosion fatigue tests in air and in the synthetic exhaust gas condensate K2.2 according to VDA 230-214 demonstrated the important role of corrosive environments for the mechanical performance of brazed stainless steel joints. Based on the results of the current study, the following conclusions could be drawn for the investigated brazed AISI 304L/BAu-4 joints.

1. For the as-received brazed specimens, a fatigue strength of 397 MPa at 2 × 10^6^ cycles was determined. The S-N curve and the cyclic deformation behaviour of the brazed joints were characteristic for the metastable austenitic base material.
2. Corrosion fatigue loading in a synthetic condensate led to a significant degradation of the fatigue properties, with a reduction of the fatigue strength at 2 × 10^6^ cycles down to 51% (202 MPa). Since corrosion mechanisms are known to be time-dependent, test frequencies that are representative of service conditions have to be investigated.
3. Strain, electrical, magnetic, temperature and electrochemical measurement techniques within fatigue and corrosion fatigue tests are well applicable to characterise the cyclic deformation and damage behaviour of the brazed joints.
4. In the synthetic condensate, a single fatigue crack initiation was environmentally assisted. After six weeks of pre-corrosion, the corroded diffusion zones facilitated several fatigue crack initiations. As different microstructure-related damage mechanisms were identified, it is recommended to consider both fatigue tests after pre-corrosions and corrosion fatigue tests for the design of brazed components.

## Figures and Tables

**Table 1:** Chemical composition of the AISI 304L base material (wt.%).

C	Cr	Ni	Mn	Si	P	S	N	Fe
0.018	18.23	8.06	1.05	0.42	0.035	0.027	0.074	bal.
